# Myocardial Defect Detection Using PET-CT: Phantom Studies

**DOI:** 10.1371/journal.pone.0088200

**Published:** 2014-02-05

**Authors:** Eugene S. Mananga, Georges El Fakhri, Joshua Schaefferkoetter, Ali A. Bonab, Jinsong Ouyang

**Affiliations:** 1 Center for Advanced Medical Imaging Sciences, Division of Nuclear Medicine and Molecular Imaging, Massachusetts General Hospital, Boston, Massachusetts, United States of America; 2 Department of Radiology, Harvard Medical School, Boston, Massachusetts, United States of America; 3 A*STAR-NUS Clinical Imaging and Research Centre (CIRC), Singapore, Singapore; NIH, United States of America

## Abstract

It is expected that both noise and activity distribution can have impact on the detectability of a myocardial defect in a cardiac PET study. In this work, we performed phantom studies to investigate the detectability of a defect in the myocardium for different noise levels and activity distributions. We evaluated the performance of three reconstruction schemes: Filtered Back-Projection (FBP), Ordinary Poisson Ordered Subset Expectation Maximization (OP–OSEM), and Point Spread Function corrected OSEM (PSF–OSEM). We used the Channelized Hotelling Observer (CHO) for the task of myocardial defect detection. We found that the detectability of a myocardial defect is almost entirely dependent on the noise level and the contrast between the defect and its surroundings.

## Introduction

The utility of ^13^N-ammonia, ^15^O-water, and ^82^Rb as PET perfusion tracers has been well documented in the cardiac PET community [*1*], [*2*]. Furthermore, ^18^F-BMS-747158 [*2*]–[*5*] has the potential to replace SPECT for perfusion studies. In addition, ^18^F-fluorodeoxyglucose (FDG) uptake in the myocardium has been well validated as an indicator of myocardial viability [*6*]. In the clinical setting, diagnosis of cardiac disease is often strongly correlated with the detectability of a defect, which can be affected by noise level as well as activity distribution. Therefore, it is important to investigate defect detectability under different injection doses/imaging times and activity distributions. Myocardial defect detectability can be impacted by the activity distribution in both the heart and other organs or tissues surrounding the heart. It would be very difficult to use clinical data to study such impact.

Lesion detection is an important clinical task for medical imaging. In oncology, lesion detectability is critically important for early diagnosis and staging of patients. Typically, lesion detectability pertains to hot lesions (e.g., tumors, inflammation), where the uptake is higher within the lesions as compared to their surrounding background [*7*], [*8*]. Previous research on myocardial defect detection using PET is limited. Tang *et al*. [*9*] studied defect detection using ^82^Rb-PET Monte Carlo simulation. Other myocardial defect detection results are reported on SPECT imaging by Chen *et al.* [*10*], Wollenweber *et al.* [*11*], and Matsunari *et al.* [*12*].

In this paper, we propose a methodology to study myocardial defect detectability using phantom studies on a PET–CT scanner. We performed cardiac phantom studies to characterize myocardial defect detectability as a function of total number of counts, which is related to noise level, and variable activity distributions. We modeled defect-present and defect-absent studies and used the Channelized Hotelling Observer (CHO) [*13*]–[*15*] as a surrogate of human observer performance. Observer SNR was evaluated in the task of defect detection in a signal–known–exactly/background–known–exactly (SKE-BKE) paradigm with three types of image reconstruction: Filtered Back-Projection (FBP), Ordinary Poisson–Ordered Subsets Expectation Maximization (OP–OSEM), and Point Spread Function corrected OSEM (PSF–OSEM).

## Materials and Methods

### 1. Data acquisitions

**Figure 1:**
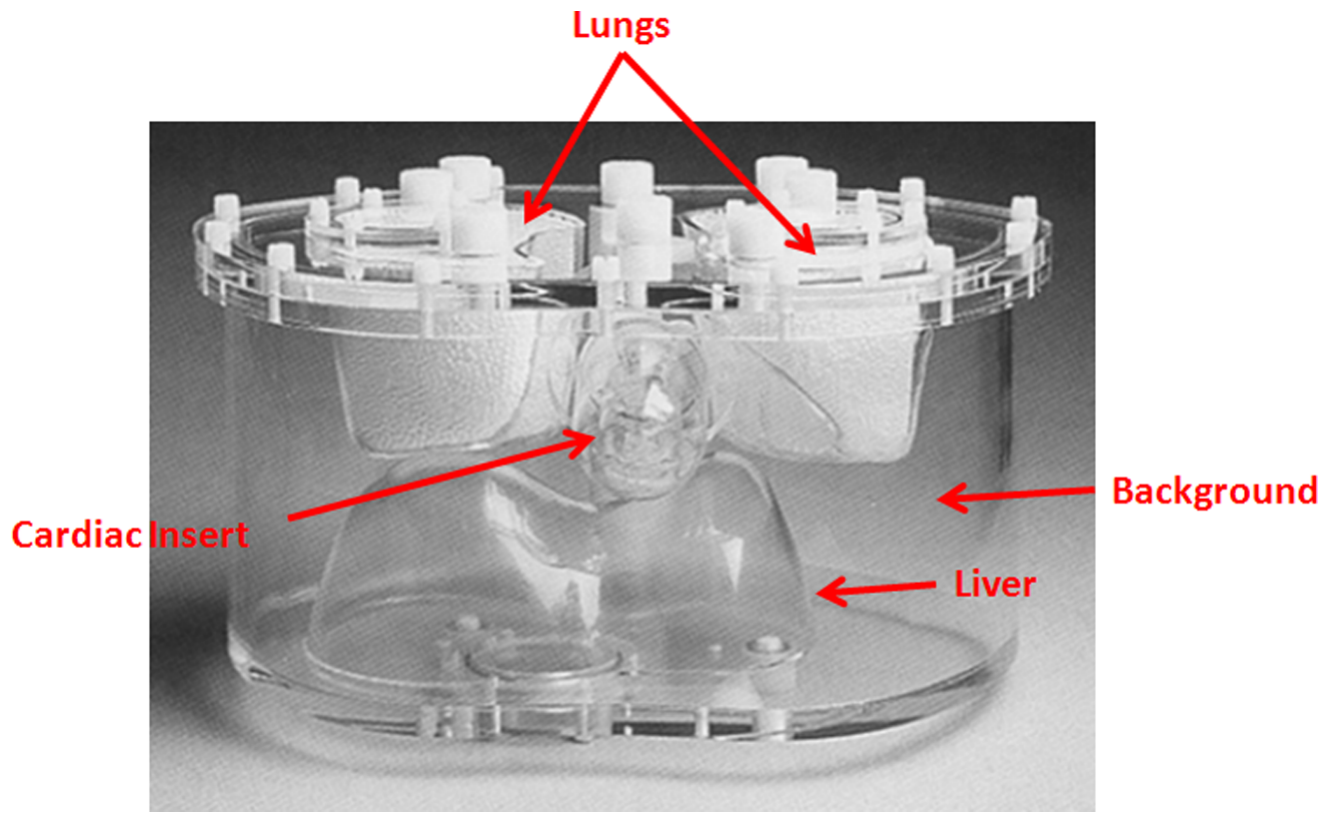
The anthropomorphic torso phantom used for the study.

An anthropomorphic torso phantom (Data Spectrum, Hillsborough, NC) with a cardiac insert was used for all the acquisitions (See Fig. 1). The phantom simulates upper torso of average to large male/female subjects (38×26 cm). This phantom included heart (myocardium wall, defect, and myocardium cavity), left and right lungs, liver, spine, and a soft tissue compartment. The fillable defect placed within the myocardium cavity has 45°×2 cm with 3.8 mL volume. All the phantom experiments were performed on a Siemens Biograph PET–CT at Massachusetts General Hospital (MGH). Virtually noise-free ^18^F data were acquired for the following five data sets:

1. *Myocardium*: The myocardium wall was filled with radioactive water while the defect, the liver, the lung, and the soft tissue background compartments were filled with “cold” water.
2. *Defect*: The defect compartment was filled with radioactive water and all other compartments were filled with “cold” water.
3. *Liver*: The liver compartment was filled with radioactive water and all other compartments were filled with “cold” water.
4. *Lung*: The lung compartments were filled with radioactive water and all other compartments were filled with “cold” water.
5. *Background*: The soft tissue background compartment was filled with radioactive water while the liver, the lung, the defect, and the myocardium wall compartments were filled with cold water.

For all the acquisitions, the phantom was always placed at the same position on the bed of the scanner.

### 2. Data set combinations

**Figure 2:**
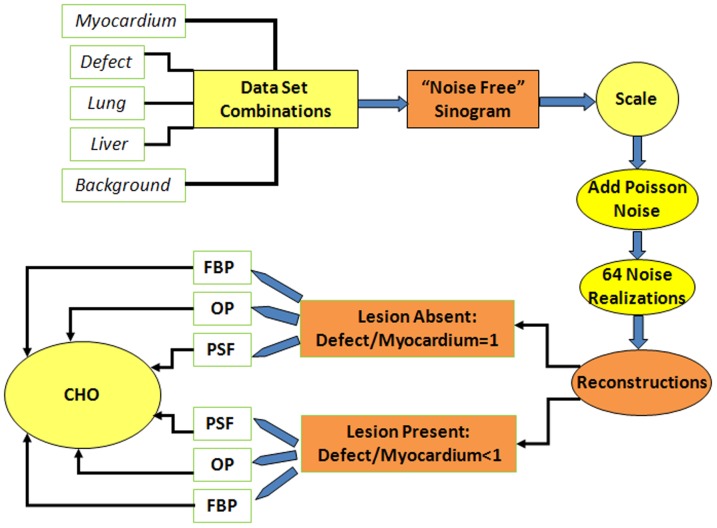
Flow chart of the data set combination, reconstruction, and CHO analysis.

Fig. 2 shows the flow chart of data set combinations, image reconstruction, and the analysis of defect detection. From *myocardium, defect, liver, lung, and background* data sets, we generated various activity combinations. For each activity combination, we created two virtually “noise-free” sinogram data sets: defect-absent and defect-present. Each original data set was scaled before mixing to account for the radioactive decay, scan time, and injected activity (measured by a dose calibrator). The synthesized sinogram data sets were then scaled again to yield total 20 million counts in the soft-tissue background. We then added Poisson noise to the “noise-free” sinogram data sets to generate 64 noise realizations. The acquired CT images of the phantom were used for accurate modeling of attenuation and scatter media during the image reconstruction.

**Figure 3:**
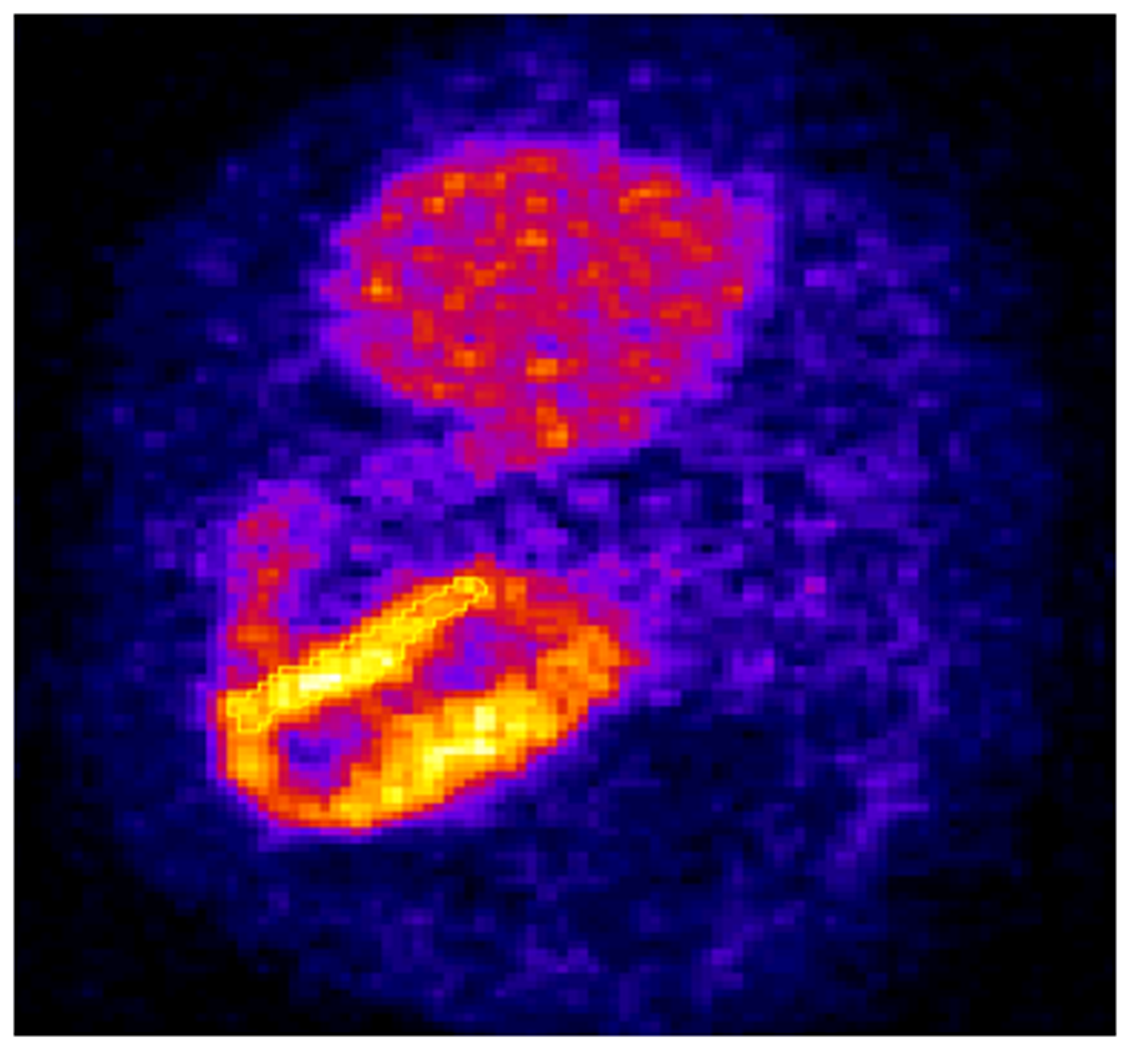
A reconstructed image slice through the myocardium for an ^18^F-BMS747158 patient study.

In order to combine *myocardium, defect, liver, lung, and background* data sets to generate a realistic activity distribution, we first investigated a cardiac PET patient study with ^18^F-BMS747158 flow agent (see Fig. 3). The patient study was approved by Partners Institutional Review Board (IRB). The patient study showed that the myocardium-to-background, liver-to-background, lung-to-background, and defect-to-myocardium concentration ratios were 11.14, 6.02, 0.63, and 0.825, respectively. The total number of detected coincidence events from the soft-tissue background was around 20 million. These concentration ratios and total number of counts were used as the default for our phantom studies except the parameter under investigation.

### 3. Reconstructions

Three reconstruction schemes were considered: FBP, OP-OSEM, and PSF-OSEM. The image reconstruction was performed using Siemens e7-tools, which incorporated attenuation and scatter corrections using the acquired CT map of the phantom. For both OP-OSEM and PSF-OSEM, we used 2 iterations and 8 subsets unless stated otherwise. No post-reconstruction filtering was applied. All the image reconstruction was performed using 168×168×81 matrix size and 4.07×4.07×2.02 mm voxel size.

### 4. Observer Model

The acquisition and processing schemes were assessed on the basis of performance of a model observer in detecting the presence of a defect of known position. The CHO, which has been shown to have a good agreement with human performance [*16*], is the numerical observer used in this work. The model used a 3-channel Hotelling observer, in which the 168×168×81 image volume data were processed through the frequency channels that are believed to exist in the human visual system. Sixty four defect-present and 64 defect-absent noise realizations were used to compute the 3–dimensional CHO SNR for myocardial defect detection. The CHO–SNR is given by:
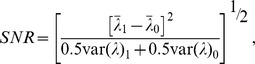
where 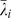 and 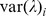 are the mean and variance (*i = 1* for defect-present; *i = 0* for defect-absent), respectively. We used the CHO with sparse difference–of–Gaussian (S–DOG) channel configuration (three radially symmetric channel profiles). The DOG channels are defined by:
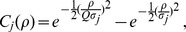
where 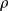 is the radial frequency, 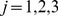 is the channel index, and 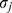 is defined by:
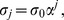
where 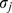 is the variance of the two Gaussian curves in frequency domain. The channel parameters used were 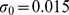, 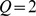, and 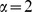.

Theoretically, a channel model is used to reduce the dimensionality of the data. In our study, we chose the above system of radially symmetric channels for simplification. This type of CHO reduces the dimensionality of the covariance matrix to the number of channels by the number of channels. For instance, in our case, for the image volume 168×168×81, the covariance matrix size is of 2.3 M×2.3 M. Using the three channel model, the covariance matrix reduces to 3×3×3. This massive reduction in dimensionality of the covariance matrix allows for the estimation problem to be tractable with a reasonable sized data set of images.

Overall, Fig. 2 shows the flow chart used to combine data, scale data, create noise realizations, reconstruct data for both lesion-absent and lesion-present cases, and perform CHO analysis.

## Results and Discussion

**Figure 4:**
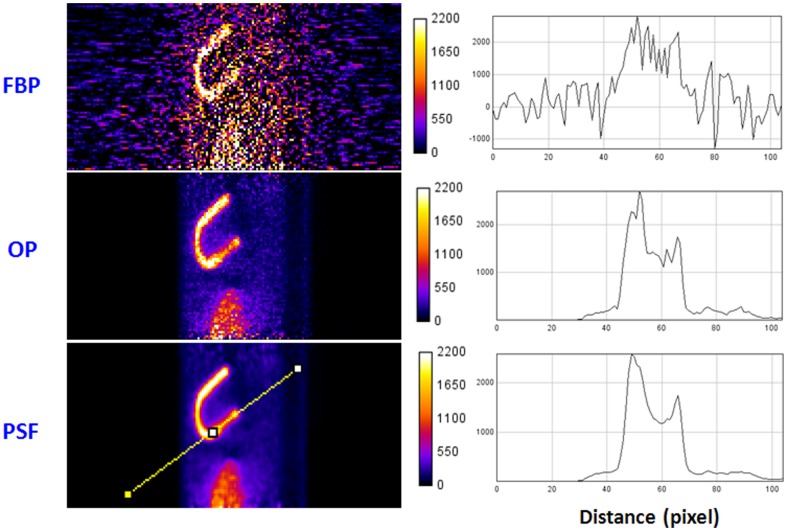
Reconstructed image slices and line profiles through the myocardial defect.

Fig. 4 shows one reconstructed slice through the myocardial defect for the default case described in Sec. 2, Materials and Methods. As expected, the image reconstructed by FBP appears noisier and has lower contrast than images reconstructed by iterative algorithms. PSF-OSEM yields almost the same contrast as compared to OP-OSEM.

**Figure 5:**
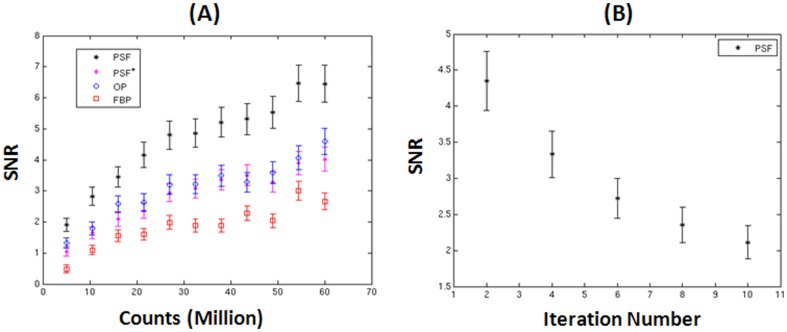
CHO SNR versus total number of counts in the background (A) and iteration number using PSF-OSEM for the default case described in Sec. 2, Materials and Methods.

Fig. 5(A) shows CHO SNR versus total number of background counts ranging from 5 to 60 million for each reconstruction algorithm. As expected, the SNR increases as the total number of background counts increases. The SNR value was found to be approximately proportional to the square root of the number of total counts. For each total number of counts, PSF-OSEM reconstruction was performed twice; one with 2 iterations and 8 subsets (denoted PSF-OSEM) and the other with 8 iterations and 8 subsets (denoted PSF^+^-OSEM). The CHO SNRs for both OP-OSEM and PSF^+^-OSEM are almost the same. PSF^+^-OSEM was found to yield almost the same noise level in the background region as OP-OSEM. This implies that PSF^+^-OSEM yields almost the same defect contrast as OP-OSEM. Although PSF modeling is expected to correct partial volume effect (PVE) and improve defect contrast, the improvement is negligible for the defect we used, which has relatively large size. The reason that PSF-OSEM yields higher SNR as compared to OP-OSEM is mostly due to the fact the noise level produced by PSF-OSEM is lower than OP-OSEM at the same iteration number because PSF-OSEM converges more slowly than OP-OSEM. Fig. 5(B) shows that SNRs decreases as the number of iterations increases for the default case using eight subsets. As the number of iterations increases, SNR decreases due to increased noise level.

**Figure 6:**
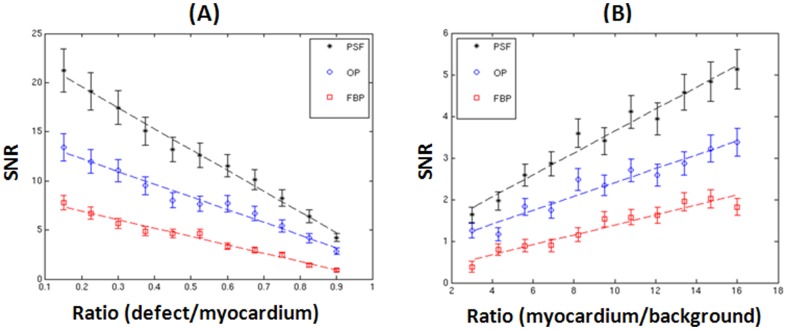
CHO SNR versus defect/myocardium (A) and myocardium/background (B) concentration ratios. The dashed lines were obtained using weighted least squares linear regression.

Fig. 6(A) shows CHO SNR versus defect/myocardium activity concentration ratio ranging from 0.15 to 0.9. The coefficients of determination (*R* ^2^) for the least squares linear regression lines are: 0.978, 0.976, and 0.989 for FBP, OP-OSEM, and PSF-OSEM, respectively. The defect detectability increases as the defect/myocardium decreases. The SNR value vanishes as defect/myocardium approaches 1, i.e., defect-absent myocardium. Fig. 6(B) illustrates the defect detectability as a function of myocardium/background concentration ratio. The *R* ^2^ coefficients are: 0.926, 0.939, and 0.969 for FBP, OP-OSEM, and PSF-OSEM, respectively. Because we fixed defect/myocardium ratio to be 0.825 for this study, defect/background ratio increases as myocardium/background increases. That results in increased SNRs, which is partially determined by the contrast of the defect relative to its surrounding region, as myocardium/background increases.

**Figure 7:**
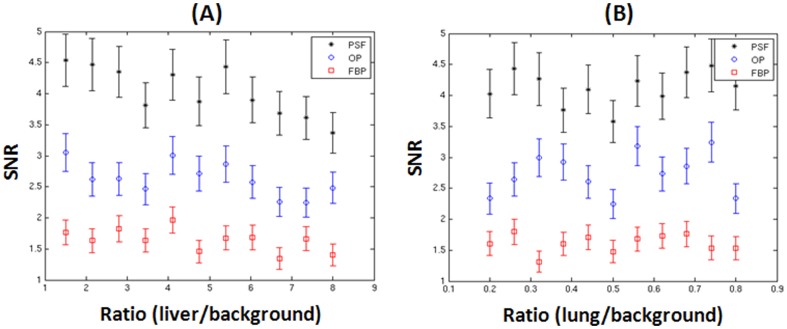
CHO SNR versus liver/background (A) and lung/background (B) concentration ratios.

Fig. 7(A) shows CHO SNR versus the activity concentration ratio of liver/background ranging from 1.5 to 8. This figure shows that the SNR is relatively insensitive to the liver/background concentration ratios. SNR decreases slightly as the concentration ratio liver/background increases from 1.5 to 8. On the other hand, Fig. 7(B) shows CHO SNR versus the activity concentration ratio of lung/background ranging from 0.2 to 0.8. This figure also illustrates that the SNR is insensitive to the lung/background activity concentration ratios for all the data points.

For all the studies, FBP always yields the lowest CHO SNRs. For the same iteration number and the same number of subsets, PSF-OSEM always yields the higher CHO SNR than OP-OSEM.

## Conclusions

Defect detectability was assessed as a function of total number of background counts (noise level), defect/myocardium, myocardium/background, liver/background, and lung/background concentration ratios. As expected, the SNR increases as the noise level decreases. The SNR also increases as the defect/myocardium concentration ratio decreases. The results show that the SNR is relatively insensitive to the liver/background and lung/background concentration ratios. FBP yields the lowest CHO SNRs as compared to iterative reconstruction algorithms. Also, PSF-OSEM yields higher CHO SNRs than OP-OSEM for the same iteration number and same number of subsets. However, PSF-OSEM and OP-OSEM yield almost the same CHO SNR if noise levels are the same. This is mainly due to the fact that the defect size we used was relatively big.

Our phantom study shows that the detection SNR is dominated by the contrast of defect to its surrounding background and the noise level. The study of defect detection SNR as function of total number of coincidence counts can be useful to optimize injection dose and/or imaging time. The studies of defect detection versus various activity distributions in the lungs and liver prove that activity uptake in the liver and lungs will not have a major impact on the detection of a myocardial defect. In summary, this phantom study shows that the detectability of a myocardial defect is dominated by the noise level and the contrast between the defect and its surroundings. Therefore, accurate identification of a myocardial defect can be achieved using PET regardless of activity distribution.
